# Sentinel Lymph Node Biopsy in Patients with Thick Primary Cutaneous Melanoma: Patterns of Use and Underuse Utilizing a Population-Based Model

**DOI:** 10.1155/2013/315609

**Published:** 2013-01-10

**Authors:** Steve R. Martinez, Dhruvil R. Shah, Anthony D. Yang, Robert J. Canter, Emanual Maverakis

**Affiliations:** ^1^Division of Surgical Oncology, Department of Surgery, University of California at Davis, Saeramento, CA 95817, USA; ^2^UC Davis Comprehensive Cancer Center, 4501 X Street, Suite 3010, Sacramento, CA 95817, USA; ^3^Department of Dermatology, University of California at Davis, Saeramento, CA 95817, USA; ^4^Department of Veteran Affairs, Northern California Health Care System, Sacramento, CA 95655, USA

## Abstract

*Background*. Sentinel lymph node biopsy (SLNB) for thick cutaneous melanoma is supported by national guidelines. We report on factors associated with the use and underuse of SLNB for thick primary cutaneous melanoma. *Methods*. The Surveillance, Epidemiology, and End Results database was queried for patients who underwent surgery for thick primary cutaneous melanoma from 2004 to 2008. We used multivariate logistic regression models to predict use of SLNB. *Results*. Among 1,981 patients, 833 (41.8%) did not undergo SLNB. Patients with primary melanomas of the arm (OR 2.07, CI 1.56–2.75; *P* < 0.001), leg (OR 2.40, CI 1.70–3.40; *P* < 0.001), and trunk (OR 1.82, CI 1.38–2.40; *P* < 0.001) had an increased likelihood of receiving a SLNB, as did those with desmoplastic histology (OR 1.47, CI 1.11–1.96; *P* = 0.008). A decreased likelihood of receiving SLNB was noted for advancing age ≥ 60 years (age 60 to 69: OR 0.58, CI 0.33–0.99, *P* = 0.047; age 70 to 79: OR 0.32, CI 0.19–0.54, *P* < 0.001; age 80 or more: OR 0.10, CI 0.06–0.16, *P* < 0.001) and unknown race/ethnicity (OR 0.21, CI 0.07–0.62; *P* = 0.005). *Conclusions*. In particular, elderly patients are less likely to receive SLNB. Further research is needed to assess whether use of SLNB in this population is detrimental or beneficial.

## 1. Introduction

Lymphatic mapping and sentinel lymph node biopsy (SLNB) was developed by Morton et al. as an alternative to elective lymph node dissection for patients with intermediate thickness melanoma [1]. However, information from institutional studies and post hoc analyses from randomized clinical trials indicate that SLNB provides accurate and important prognostic information, even among patients with thick melanoma [2–11]. Current guidelines from the National Comprehensive Cancer Network (NCCN) advocate the SLNB for all melanomas >1 mm in thickness [12]. Such guidelines have been in place since 1998 [13]. 

The use of SLNB for thick cutaneous melanomas has nevertheless remained controversial due to the nihilistic belief that thick melanomas are associated with an unacceptably high likelihood of distant metastatic disease [14]. Given that only half of all patients with thick primary melanomas will be alive 10 years after their diagnosis, [2] some physicians believe that such patients are unlikely to benefit from SLNB because their outcome will be dictated by their occult metastasis and not the presence or absence of sentinel lymph node metastasis.

To date, little data have been acquired regarding use of SLNB for thick melanomas, and the factors that may contribute to its use are largely unknown. Our goals were to assess the prevalence of SLNB use for thick primary cutaneous melanoma and to identify clinical and pathologic factors associated with its use or underuse utilizing a national, population-based database.

## 2. Methods

We used the Surveillance, Epidemiology, and End Results (SEER) database to identify patients diagnosed with thick (known Breslow depth > 4.00 mm) primary cutaneous melanoma treated surgically from 2004 to 2008. These years were selected because they were subsequent to reports in the surgical and oncology literature supportive of SLNB for thick primary cutaneous melanoma and guidelines established in 1998 [13]. The registries, attributes, and limitations of the SEER database have been reported previously [15]. Because SEER data are de identified, this research was exempt from Institutional Review Board approval.

We excluded patients with mucosal melanoma, those without a biopsy-proven diagnosis, those diagnosed at autopsy, patients whose lymph node evaluation was unknown, or other than SLNB “yes” or SLNB “no”. Patients who had a lymph node evaluation other than SLNB were also excluded, as it was assumed that these patients likely had clinically evident, palpable disease and underwent formal complete lymphadenectomy. 

To assess the proportion of patients with thick primary melanomas undergoing SLNB over time, we tabulated the number of SLNB procedures performed relative to the total number of cases of thick primary cutaneous melanoma per year. 

We used multivariate logistic regression to predict use of SLNB. Covariates examined in the model included patient age (ages 1 to 39; 40 to 49; 50 to 59; 60 to 69; 70 to 79; 80 or more), sex, race/ethnicity (Asian, black, Hispanic, and white), Breslow depth (continuous variable per 0.01 mm), tumor histology (acral lentiginous, amelanotic, desmoplastic, epidermoid, lentigo maligna, nevus associated, nodular, regressing, and superficial spreading), tumor location (arm, head/neck, leg, trunk, and overlapping), and ulceration status (yes, no, and unknown). The likelihood of undergoing SLNB was reported as odds ratios (OR) with 95% confidence intervals (CI). Significance was set at *P* ≤ 0.05. All statistical analyses were performed using STATA 11 (College Station, TX).

## 3. Results

Among 1,981 patients with thick primary cutaneous melanoma, 833 (41.8%) did not undergo SLNB. The majority of patients were white (91.7%), male (63.6%), and elderly, with approximately 70% of patients being age 60 or older. The mean Breslow thickness of the primary cutaneous melanomas in this group of patients with thick melanomas was 6.55 mm. Tumor ulceration was noted in 54.7% of cases. Melanomas were mostly of nodular (52.7%), desmoplastic (19.3%), and superficial spreading (14.9%) histology. Head/neck, arm, and truncal tumor sites were almost equally represented (28.9%, 28.2%, and 25.3%, resp.). We looked at the proportion of patients receiving SLNB for their thick cutaneous melanoma by year of diagnosis. There was a general trend towards increasing utilization of SLNB, with 50.8% of patients having the procedure in 2004, 58% in 2005, 56.4% in 2006, 63.5% in 2007, and 61.1% having it in 2008.

Patient and tumor characteristics of the study population according to whether or not a SLNB was performed are included in Table 1. When divided into those that received a SLNB and those that did not receive a SLNB, the two groups were found to significantly differ with respect to patient age, mean Breslow tumor depth, and tumor location. Those in the no SLNB group had a greater proportion of elderly patients (80 years or older), thinner melanomas, and a greater proportion of head/neck tumor sites than those in the SLNB group. 

Results of the multivariate logistic regression analysis are presented in Table 2. Factors associated with a decreased likelihood of receiving a SLNB included age 60 to 69 (OR 0.58, CI 0.33–0.99; *P* = 0.047), 70 to 79 (OR 0.32, CI 0.19–0.54; *P* < 0.001), 80 years or older (OR 0.10, CI 0.06-0.16, *P* < 0.001), and unknown race/ethnicity (OR 0.21, CI 0.07–0.62, *P* = 0.005). Factors associated with an increased likelihood of receiving a SLNB included primary melanomas of the arm (OR 2.07, CI 1.56–2.75; *P* < 0.001), leg (OR 2.40, CI 1.70–3.40; *P* < 0.001), and trunk (OR 1.82, CI 1.38–2.40; *P* < 0.001), as well as desmoplastic histology (OR 1.47, CI 1.11–1.96; *P* = 0.008). 

## 4. Discussion

Despite the fact that SLNB for lymph node staging of thick primary cutaneous melanomas has been advocated by national guidelines since 1998 [13] and supported by retrospective and prospective institutional data [2–11], its use in this setting is frequently cited as controversial. While we support the use of SLNB as a staging tool for patients with thick primary cutaneous melanoma, we understand that some surgeons have a bias against its use in this setting. Our goals were to identify the proportion of patients with thick melanomas undergoing SLNB and to assess patient and tumor-related factors that may contribute to the use or underuse of SLNB.

In our current study of 1,981 patients with thick primary cutaneous melanoma, 41.8% did not have a SLNB. This is higher than the 19.5% underuse rate reported by Meguerditchian et al. Most in an institutional review of 113 similar patients [16]. There are several possible explanations for this discrepancy. Among them is an important limitation of the SEER database. SEER does not provide information on individual patient comorbidities that may influence the recommendation to perform a SLNB. For example, a surgeon may be more likely to perform a SLNB on a healthy 80-year-old man than a similarly aged man with diabetes, congestive heart failure, and exercise limiting chronic obstructive pulmonary disease, but SEER cannot shed light on this aspect of the treatment decision-making process. 

We found that the likelihood of receiving a SLNB was inversely proportional with age, with patients aged 60 years and older having a decreased likelihood of undergoing a SLNB. This finding is consistent with our previously reported finding that elderly patients are less likely to receive SLNB for intermediate thickness melanoma [17]. Older patients are more likely than their younger counterparts to have comorbidities that may influence the decision of whether or not to offer SLNB. Significant comorbidities, when added to the risk of distant metastasis and subsequent mortality from a thick primary cutaneous melanoma, may be perceived to overshadow any potential benefit obtained from the SLNB in terms of accurate staging and early complete lymphadenectomy in the case of node positive disease. Poorer overall survival among elderly patients is to be expected. However, Göpper et al. found that, eventhough increasing age was associated with decreased overall survival, this effect was dominated by the prognostic importance of the sentinel lymph node status [18]. Among the largest single institution series was reported by Gajdos et al. [19]. They examined 227 patients with thick (T4) melanoma who underwent SLNB from 1997 to 2007 and similarly found that increasing age negatively influenced overall survival. Again, however, the influence of advancing age was dominated by the status of the sentinel lymph node. When patients were further categorized into sentinel lymph node negative and sentinel lymph node positive groups, increasing age was found to be a prognostic factor only for the sentinel lymph node negative group. Together, these findings indicate that, even as age increases, the sentinel node status may still provide valuable prognostic information. 

In our study, we noted that patients of unknown race/ethnicity were significantly less likely to undergo SLNB. We have no definitive explanation for this finding. Our patient population was > 90% white, with very few (*N* = 16) being of unknown race/ethnicity. It is most likely that this finding is unreliable for this reason.

Increasing Breslow thickness negatively influenced the likelihood of receiving a SLNB in our study. Our multivariate model assessed the likelihood of receiving a SLNB for each 0.01 mm increase in Breslow thickness. For every 0.01 mm increase in Breslow depth, there was a 0.5% decrease in the odds of receiving a SLNB. This translates into a 14.5% decrease in the odds of receiving a SLNB for every 1 mm increase in Breslow thickness. This is likely explained by a growing sense of nihilism about the likelihood of occult distant metastatic disease as the primary tumor increases in thickness. It is somewhat unclear if the nihilism is warranted, however. Carlson et al. studied 114 patients with thick melanoma who underwent SLNB and found that the thickest tumors (>6 mm) were not associated with increased risks of overall or relapse-free survival [20]. Using tumor thickness as a continuous variable in a multivariate model, Gutzmer et al. failed to identify any influence of increasing melanoma thickness on overall survival [6]. Similarly, Gershenwald et al. noted no influence of increasing tumor thickness on either disease-free or overall survival among 131 patients with thick melanomas undergoing SLNB [5]. Both Ferrone et al. [4] and Jacobs et al. [7] documented a higher risk of disease recurrence with increasing tumor thickness, but they did not examine any influence on overall survival. 

We found that patients with thick desmoplastic melanomas were less likely to undergo SLNB. It is possible that SLNB was less commonly offered in these patients because desmoplastic melanomas have been shown to have lower rates of sentinel lymph node metastasis [21]. Additionally, desmoplastic melanomas are more common on the head/neck, and these areas may be more challenging for lymphatic mapping and SLNB may therefore be deferred in favor of clinical observation. Indeed, in our analysis, relative to head/neck primary sites, patients with melanomas of the trunk and extremities were approximately twice as likely to undergo SLNB.

Although the presence of tumor ulceration plays a significant role in several models assessing the prognostic utility of SLNB in patients with thick melanoma, [4, 5, 7, 20] ulceration did not seem to influence the use of SLNB in our patient cohort. This could reflect a relationship between tumor ulceration and Breslow thickness. As previously stated, we found that increasingly thick melanomas were less likely to undergo SLNB. Due to tumor ulceration, the depth of some melanomas may be underestimated. With these “thinner” melanomas being perceived as “less risky” for occult metastasis, surgeons may opt to pursue SLNB more readily. However, Bilimoria et al. examined the records of 8,525 stage IB and II melanoma patients in the National Cancer Data Base and found that those with no tumor ulceration were less likely to undergo SLNB [22].

Our data may be subject to selection bias, since we only included patients for whom data was complete regarding whether or not SLNB was performed. Such a decision provided us with a more homogeneous population and allowed us to exclude patients that underwent a complete lymphadenectomy, likely because of clinically palpable nodal disease. Nevertheless, we may have excluded patients that would have otherwise altered our measured outcomes. Finally, SEER does not provide information on patient-level socioeconomic status such as monthly/yearly income or surrogates of socioeconomic status like education level or insurance status. Such factors may influence access to optimal cancer care and may partially explain the relatively high percentage of patients not undergoing SLNB in our study.

 SLNB for patients with thick primary cutaneous melanoma should no longer be considered controversial. Despite this, a significant proportion of patients do not undergo lymph node staging with SLNB. We have demonstrated that patients age 60 years and older are less likely to receive SLNB. To date, data do not suggest that limiting SLNB in the elderly is appropriate. Further research to assess whether use of SLNB in older patients is detrimental or beneficial is needed.

## Figures and Tables

**Table 1:** Patient and tumor characteristics of 1,991 patients with thick primary cutaneous melanoma according to whether or not they received a sentinel lymph node biopsy (SLNB).

Variable	SLNB, *N* = 1,158 (%)	No SLNB, *N* = 833 (%)	*P* value
Age, years			
1 to 39	97 (8.4)	21 (2.5)	<0.001
40 to 49	135 (11.7)	43 (5.2)	
50 to 59	239 (20.6)	65 (7.8)	
60 to 69	243 (21)	97 (11.6)	
70 to 79	268 (23.1)	192 (23.1)	
80 or more	176 (15.2)	415 (49.8)	
Sex			
Female	416 (35.9)	525 (63)	=0.631
Male	742 (64.1)	308 (37)	
Race/ethnicity			
Asian	22 (1.9)	8 (1)	=0.062
Black	13 (1.1)	14 (1.7)	
Hispanic	55 (4.8)	30 (3.6)	
Native American	6 (0.5)	1 (0.1)	
Unknown	6 (0.5)	10 (1.2)	
White	1,056 (91.2)	770 (92.4)	
Mean Breslow depth (mm)	6.83	6.35	<0.001
Tumor histology			
Acral lentiginous	58 (5)	26 (3.1)	=0.053
Amelanotic	33 (2.9)	19 (2.3)	
Desmoplastic	225 (19.4)	160 (19.2)	
Epidermoid	28 (2.4)	16 (1.9)	
Lentigo maligna	16 (1.4)	23 (2.8)	
Nevus associated	7 (0.6)	4 (0.5)	
Nodular	606 (52.3)	463 (55.6)	
Regressing	9 (0.8)	1 (0.1)	
Superficial Spreading	176 (15.2)	121 (14.5)	
Tumor location			
Arm	301 (26)	202 (24.3)	<0.001
Head/neck	261 (22.5)	315 (37.8)	
Leg	226 (19.5)	114 (13.7)	
Trunk	368 (31.8)	194 (23.3)	
Overlapping/other	2 (0.2)	8 (1)	
Tumor ulceration			
No	501 (43.3)	356 (42.7)	=0.857
Yes	632 (54.6)	456 (54.7)	
Unknown	25 (2.2)	21 (2.5)	

**Table 2:** Multivariate logistic regression model of the entire cohort (2004–2008) predicting use of sentinel lymph node biopsy (SLNB).

Variable	Odds Ratio (95% CI)	*P* value
Age, years		
1 to 39	Referent	Referent
40 to 49	0.74 (0.41–1.35)	=0.325
50 to 59	0.84 (0.48–1.47)	=0.547
60 to 69	0.58 (0.33–0.99)	**=0.047**
70 to 79	0.32 (0.19–0.54)	**<0.001**
80 or more	0.10 (0.06–0.16)	**<0.001**
Sex		
Male	Referent	Referent
Female	0.93 (0.75–1.16)	=0.532
Race/ethnicity		
White	Referent	Referent
Asian	1.54 (0.61–3.91)	=0.359
Black	0.54 (0.23–1.27)	=0.158
Hispanic	0.85 (0.51–1.41)	=0.523
Native American	4.62 (0.45–47.63)	=0.198
Unknown	0.21 (0.07–0.62)	**=0.005**
Mean Breslow depth (per 1/100 mm)	0.99 (0.99–1.00)	**<0.001**
Tumor histology		
Nodular	Referent	Referent
Acral lentiginous	1.08 (0.61–1.92)	=0.786
Amelanotic	1.21 (0.64–2.30)	=0.555
Desmoplastic	1.47 (1.11–1.96)	**=0.008**
Epidermoid	1.36 (0.67–2.76)	=0.388
Lentigo maligna	0.93 (0.45–1.91)	=0.850
Nevus associated	0.91 (0.24–3.45)	=0.887
Regressing	4.83 (0.58–40.47)	=0.147
Superficial Spreading	0.91 (0.68–1.23)	=0.549
Tumor location		
Head/neck	Referent	Referent
Arm	2.07 (1.56–2.75)	**<0.001**
Leg	2.40 (1.70–3.40)	**<0.001**
Trunk	1.82 (1.38–2.40)	**<0.001**
Overlapping/other	0.23 (0.04–1.22)	=0.084
Tumor ulceration		
Yes	Referent	Referent
No	0.88 (0.71–1.09)	=0.234
Unknown	0.63 (0.33–1.22)	=0.172
